# An Overview on Medicinal Chemistry of Synthetic and Natural Derivatives of Cannabidiol

**DOI:** 10.3389/fphar.2017.00422

**Published:** 2017-06-28

**Authors:** Paula Morales, Patricia H. Reggio, Nadine Jagerovic

**Affiliations:** ^1^Department of Chemistry and Biochemistry, University of North Carolina Greensboro, GreensboroNC, United States; ^2^Instituto de Química Médica, Consejo Superior de Investigaciones Científicas, Unidad Asociada I+D+i al Instituto de Química Médica/Universidad Rey Juan CarlosMadrid, Spain

**Keywords:** cannabidiol, cannabidiol derivative, cannabinoid receptor, molecular target, therapeutic application

## Abstract

Cannabidiol (CBD) has been traditionally used in *Cannabis*-based preparation, however historically, it has received far less interest as a single drug than the other components of *Cannabis*. Currently, CBD generates considerable interest due to its beneficial neuroprotective, antiepileptic, anxiolytic, antipsychotic, and anti-inflammatory properties. Therefore, the CBD scaffold becomes of increasing interest for medicinal chemists. This review provides an overview of the chemical structure of natural and synthetic CBD derivatives including the molecular targets associated with these compounds. A clear identification of their biological targets has been shown to be still very challenging.

## Introduction

**FIGURE 1:**
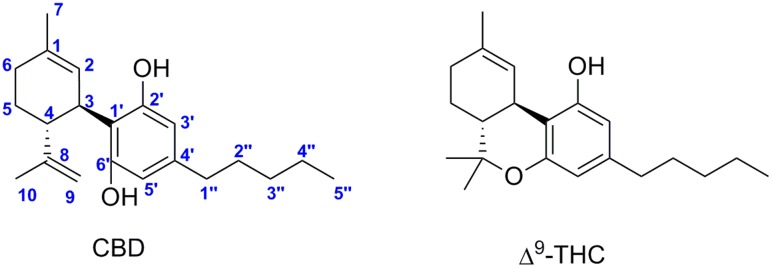
Cannabidiol (CBD) and (–)-*trans*-Δ^9^–tetrahydrocannabinol (Δ^9^–THC).

In the mid-seventies, major efforts were focused on the identification of new natural cannabinoids isolated from preparations of *Cannabis sativa* and of other subspecies and varieties, such as *Cannabis indica* and *Cannabis ruderalis*. The two most abundant and most therapeutically relevant components of the plants are (–)-*trans*-Δ^9^–tetrahydrocannabinol (Δ^9^–THC) and (-)-cannabidiol (CBD) (**Figure 1**). Over these last two decades, the endocannabinoid system (ECS) related to the effects of *Cannabis sativa* has being emerging as target of pharmacotherapy showing very considerable physiological significance (Mechoulam et al., 2014). This system includes two cannabinoid receptors (CB_1_ and CB_2_) and endogenous ligands named endocannabinoids (Matsuda et al., 1990; Munro et al., 1993). CB_1_ receptor is abundant in the brain, but to a less extend in peripheral tissues. CB_2_ receptor is mainly expressed in immune cells.

Δ^9^–THC is responsible for the psychoactive effects of *Cannabis sativa* mediated by the activation of CB_1_ receptor in the brain, whereas CBD is considered non-psychotropic. Currently, CBD is clinically used in association with Δ^9^–THC in a cannabis-based preparation (Sativex^®^) that contains equimolar content of both for managing neuropathic symptoms associated with multiple sclerosis (Fernandez, 2016). CBD as a single drug is currently generating considerable interest due to its beneficial neuroprotective (Fernandez-Ruiz et al., 2013; Scuderi et al., 2014; Ibeas Bih et al., 2015), antiepileptic (Devinsky et al., 2015; Wright et al., 2015), hypoxia-ischemia (Lafuente et al., 2011; Mori et al., 2017), anxiolytic (Massi et al., 2013; Schier et al., 2014), antipsychotic (Bhattacharyya et al., 2010), analgesic (Maione et al., 2011), anti-inflammatory (Ruiz-Valdepeñas et al., 2011; Burstein, 2015), anti-asthmatic (Ribeiro et al., 2015; Vuolo et al., 2015), and antitumor properties (McAllister et al., 2011; Massi et al., 2013) among others(Mechoulam et al., 2007; Zhornitsky and Potvin, 2012; Renard et al., 2017; Watt and Karl, 2017). In 2016, GW pharmaceuticals reported the first positive results of CBD (Epidiolex^®^) in phase III clinical trials for treatment-resistant seizure disorders, including Lennox–Gastaut and Dravet syndromes. An overview of regulatory approvals and clinical trials of CBD has been recently published (Fasinu et al., 2016).

The molecular targets involved in the diverse therapeutic properties produced by CBD are still not very well-understood (Morales et al., 2017). Unlike Δ^9^–THC, CBD does not bind to the orthosteric binding site of the CB_1_ and CB_2_ cannabinoid receptors (McPartland et al., 2007). Despite this lack of orthosteric affinity, CBD has been shown to antagonize the effects of the CB_1_/CB_2_ agonists CP–55,940 and WIN55212 at the mouse CB_1_ and at the human CB_2_ receptors (Pertwee et al., 2002; Thomas et al., 2007). Therefore, allosteric activity of CBD at these receptors has been hypothesized. In a recent report, CBD was shown to be a negative allosteric modulator of Δ^9^–THC and the endogenous cannabinoid 2–AG providing a possible explanation for some *in vivo* CBD effects (Laprairie et al., 2015; Morales et al., 2016). CBD has also been shown to modulate endocannabinoid tone by inhibiting the cellular uptake of the endocannabinoid anandamide (Leweke et al., 2012). This effect has been attributed to the fact that CBD competes with anandamide for binding to fatty acid-binding proteins (FABPs) which are intracellular proteins involved in the transport of anandamide to its metabolic enzyme fatty acid amide hydrolase (FAAH) (Elmes et al., 2015). Other possible molecular targets of CBD have been explored. Modulation of the GPR55 receptor by CBD has been evaluated in different signaling pathway assays. CBD acts as an antagonist preventing [^35^S]GTPγS binding and Rho activation (Ryberg et al., 2007; Whyte et al., 2009; Ford et al., 2010), modulating Ca^2+^ mobilization (Lauckner et al., 2008) and β-arrestin recruitment (Yin et al., 2009). CBD has also been proposed as an antagonist of the GPR18 cannabinoid receptor (McHugh et al., 2012, 2014). Certain actions of CBD such as anti-inflammatory and immunosuppressive effects appear to be partially mediated through the serotonin and adenosine receptors that are not considered part of the ECS. For instance, CBD acts as a full 5-HT_1A_ agonist, 5-HT_2A_ weak partial agonist and a non-competitive 5HT_3A_ antagonist (Russo et al., 2005; Yang et al., 2010; Rock et al., 2012). The ability of CBD to activate the A_1A_ adenosine receptor has also been proposed (Gonca and Darıcı, 2014). Other molecular targets have also been studied, among them, the PPARγ nuclear receptors (O’Sullivan et al., 2009; Esposito et al., 2011; Scuderi et al., 2014), glycine receptors (Ahrens et al., 2009; Xiong et al., 2012), GABA_A_ receptors (Bakas et al., 2016), and transient receptor potential (TRP) channels (De Petrocellis et al., 2011, 2012). Studies focused on the possible epigenetic regulation of skin differentiation genes by CBD revealed that CBD is a transcriptional repressor that can control cell proliferation and differentiation through DNA methylation (Pucci et al., 2013). Despite all of this data, the mechanistic bases for the effects of CBD remain complex.

Cannabidiol constitutes one of the most important components of therapeutic interest from *Cannabis sativa*. However, unlike the numerous synthesized cannabimimetics generated to provide a synthetic alternative to THC, CBD derivatives have only been superficially explored. The purpose of this review is to provide a structural overlook at natural and synthetic CBD derivatives. Due to the fact that diverse molecular targets are involved in the therapeutic properties produced by CBD, we associated CBD structures to their biological targets. Thus, this review is intended to be a useful tool especially for medicinal chemists.

The basic structure of the CBD derivatives described in this review consists of 5-alkyl resorcinols substituted in position 2 by a propenylcyclohexene. Structural modifications on the alkyl side-chain, on the propenylcyclohexene, and substitution of the phenolic hydroxyl groups are concerned. Quinone CBD analogs are also included in this classification as far as their structures are closely related to CBD.

## Natural Cannabidiol Derivatives

In a recently published review dedicated to the diversity of cannabis phytocannabinoids, the authors updated the inventory of naturally occurring CBD derivatives (Hanuš et al., 2016). Herein, we are associating these structures to their possible molecular targets.

**FIGURE 2:**
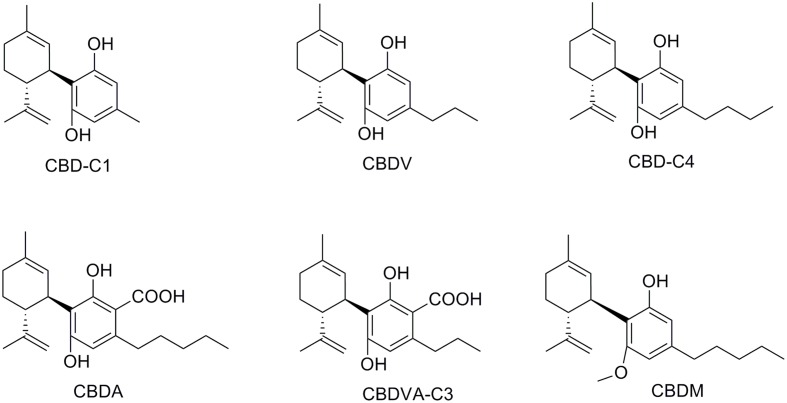
Natural phytocannabinoid CBD analogs.

Of the over 100 natural cannabinoids identified in *Cannabis Sativa*, seven have been classified as CBD-type compounds including CBD (**Figure 2**) (ElSohly and Slade, 2005; ElSohly and Gul, 2014; Aizpurua-Olaizola et al., 2016). All of them have the same absolute configuration than CBD; they are 5′-methyl-2′-(prop-1-en-2-yl)-1′,2′,3′,4′-tetrahydro-[1,1′-biphenyl]-2,6-dioles retaining the *trans*-(1*R*,6*R*) configuration. Cannabidiolic acid (CBDA) and cannabidivarinic acid (CBDVA-C3) are C3′-carboxylic derivatives, whereas cannabidiorcol (CBD-C1), cannabidiol-C4 also named as *nor*-cannabidiol (CBD-C4), and cannabidivarin (CBDV) differ from CBD by the length of their C4′-side chain. Cannabidiol monomethyl ether (CBDM), the C6′-methoxy CBD analog, was also isolated from the plant. Despite the potential therapeutic interest of these naturally occurring CBD derivatives, only a few related pharmacological studies have been reported (**Table 1**). Like most non-steroidal anti-inflammatory drugs, CBDA is characterized by a carboxylic group resulting in a selective inhibition of cyclooxygenase-2 (Takeda et al., 2008). CBDA does not have effect on anandamide inactivation in FAAH assays (Inhibition of [^14^C]-anandamide uptake: IC_50_ > 50 μM) contrary to CBD (IC_50_ = 28 μM) (Bisogno et al., 2001; Ligresti, 2006). Other molecular targets proposed for CBDA include GPR55 (Anavi-Goffer et al., 2012) and TRPA1 with moderate activity (De Petrocellis et al., 2011). CBDA has been shown to be an inhibitor of cell migration in the highly aggressive human breast cancer MDA-MB-231 by alteration of Rho GTPase activity (Takeda et al., 2012). CBDV, the C4′-propyl analog of CBD, displays very weak affinity for CB_1_ and CB_2_ receptors (Hill et al., 2013; Rosenthaler et al., 2014), whereas it has been reported to inhibit the activity of the putative endogenous ligand LPI in *h*GPR55-HEK293 cells (Anavi-Goffer et al., 2012). CBDV also targets the human TRPA1 channel (De Petrocellis et al., 2011, 2012). In several animal seizures models, CBDV exerted notable anticonvulsant effects without affecting normal motor function (Hill et al., 2012). The mechanisms through which CBDV exerts its antiepileptic effects are uncertain (Jones and Whalley, 2015). CBDV is currently in Phase II clinical trials as an antiepileptic drug under the name GWP42006.^1^

**Table 1:** CB_1_/CB_2_ cannabinoid receptor binding, molecular targets and therapeutic potential of CBD derivatives.

Compounds	CB_1_R *K*_i_ [nM]	CB_2_R *K*_i_ [nM]	Reference	Other targets	Therapeutic potential	Reference
(-)-CBD	>10000	>10000	Bisogno et al., 2001	NAM-CB1; FABPs; GPR55; GPR18; 5-HT_1A_; 5-HT_2A_; 5-HT_3A_; GlyR A_1A_; PPARγ; GABA_A_, TRPs	Neuroprotection; epilepsy; anxiety; psychosis; inflammation	Fasinu et al., 2016; Morales et al., 2017
(-)-CBDA				COX-2; TRPA1; DAGLα	Inflammation; cancer; bacteria	Appendino et al., 2008; Takeda et al., 2008, 2012; De Petrocellis et al., 2011
(-)-CBDV	14711 ± 5734	574.2 ± 146	Rosenthaler et al., 2014	GPR55; TRPA1; CYP1A1	Convulsion; epilepsy	De Petrocellis et al., 2011; Anavi-Goffer et al., 2012; Hill et al., 2012, 2013; Yamaori et al., 2013; Rosenthaler et al., 2014; Jones and Whalley, 2015
Machaeridol B	-	-		-	*Plasmodium falciparum*; *Leishmania donovani*	Muhammad et al., 2003
Ferruginene C	NR	Weak	Seephonkai et al., 2011	TRPA1	Cancer	Seephonkai et al., 2011
(-)-7-OH-CBD	>10000	>10000	Bisogno et al., 2001	-		
(+)-7-OH-CBD	5.3 ± 0.5	101.0 ± 5.1	Fride et al., 2004	-	-	
(-)-7-COOH-CBD	>10000	>10000	Bisogno et al., 2001	-	-	
(+)-7-COOH-CBD	13.2 ± 0.4	312.8 ± 15.8	Fride et al., 2004	-	-	
CBE	-	-		CYP1A1	-	Yamaori et al., 2013
(+)-CBD	842 ± 36	203 ± 16	Bisogno et al., 2001	-	-	
H_2_-CBD	>1000	-	Ben-Shabat et al., 2006	-	Inflammation	Ben-Shabat et al., 2006
H_4_-CBD	145	-	Ben-Shabat et al., 2006	-	Inflammation	Ben-Shabat et al., 2006
HU-444	>10000	>10000	Haj et al., 2015	-	Inflammation	Haj et al., 2015
HU-446	∼1000	>10000	Kozela et al., 2015	-	Inflammation	Haj et al., 2015
HU-465	76.7 ± 58	12.1 ± 2.3	Kozela et al., 2015	-	Inflammation	Haj et al., 2015
(-)-DMH-CBD	>10000	>1000	Bisogno et al., 2001	-	Anxiety; pain; inflammation; cancer	Burstein, 2015; Juknat et al., 2016
(+)-DMH-CBD	17.4 ± 1.8	211 ± 23	Bisogno et al., 2001	-	-	
(-)-7-OH-DMH-CBD	>4000	671 ± 12	Bisogno et al., 2001	TRPV1, opioid, α_2_-AR	-	Fride et al., 2005; Pertwee et al., 2005
(+)-7-OH-DMH-CBD	2.5 ± 0.03	44.0 ± 3.1	Bisogno et al., 2001	-	-	
(-)-7-COOH-DMH-CBD (HU-320)	> 1000	>4000	Bisogno et al., 2001	-	Inflammation, immunosuppression	Sumariwalla et al., 2004
(+)-7-COOH-DMH-CBD	5.8 ± 0.7	155.5 ± 5.3	Fride et al., 2004	-	-	
H_2_-DMH-CBD	124	-	Ben-Shabat et al., 2006	-		
H_4_-DMH-CBD	17	-	Ben-Shabat et al., 2006	-		
HU-308	>10000	22.7 ± 3.9	Hanus et al., 1999	-	Inflammation; hepatic ischemia; osteoporosis; pain	Ofek et al., 2006; Rajesh et al., 2007a,b; Burstein, 2015
(-)-CBDD	>10000	>10000	Hanus et al., 2005	15-LOX	Atherosclerosis, body weight regulation	Takeda et al., 2009, 2011, 2015
(+)-CBDD	>10000	>10000	Hanus et al., 2005	-	-	
(-)-DMH-CBDD	>10000	>10000	Hanus et al., 2005	-	-	
(+)-DMH-CBDD	>10000	<10000	Hanus et al., 2005	-	-	
KLS-13019	-	-			Neuroprotection	Kinney et al., 2016
HUF-101	-	-		-	Anxiety; depression, psychosis; convulsion	Breuer et al., 2016
CBD-aldehyde-diacetate	-	-		-	Convulsion	Carlini et al., 1975
6-Oxo-CBD-diacetate	-	-		-	Convulsion	Carlini et al., 1975
6-OH-CBD-triacetate	-	-		-	Convulsion	Carlini et al., 1975
9-OH-CBD-triacetate	-	-		-	Convulsion	Carlini et al., 1975
HU-331	NR	NR	Kogan et al., 2004	Topoisomerase IIα; Caspases; ROS; PARP	Cancer	Kogan et al., 2004, 2007; Petronzi et al., 2013; Regal et al., 2014
CBD-Q (V)	-	-		PPARγ	Neuroprotection	Appendino et al., 2015
CBD-Q (VIII)	-	-		PPARγ	Neuroprotection	Appendino et al., 2015
Abn-CBD	-	-		GPR55; GPR18	Vasodilatation; bacteria; diabetes; colitis	Johns et al., 2007; Ryberg et al., 2007; Appendino et al., 2008; Console-Bram et al., 2014; Krohn et al., 2016; McKillop et al., 2016
O-1602	NR	NR		GPR55; GPR18	Central nervous system; metabolism	Johns et al., 2007; Romero-Zerbo et al., 2011; Ashton, 2012; Console-Bram et al., 2014
CBG	897 ± 596	153 ± 42	Rosenthaler et al., 2014	α_2_-AR; TRP; COX-1; COX-2; 5-HT_1A_	Bacteria; bowel inflammation; depression	Appendino et al., 2008; Cascio et al., 2010; El-Alfy et al., 2010; De Petrocellis et al., 2011; Ruhaak et al., 2011; Borrelli et al., 2013; Rosenthaler et al., 2014
Cannabimovone	>10000	>10000	Taglialatela-Scafati et al., 2010	TPRV1	-	Taglialatela-Scafati et al., 2010


*NR, no response.*

**FIGURE 3:**
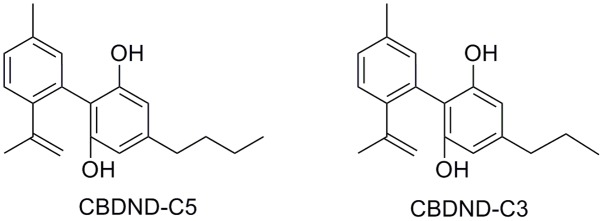
Aromatic analogs of CBD.

Two aromatic analogs of CBD have been isolated from Lebanese hashish (ElSohly and Slade, 2005): cannabinodiol (CBND-C5), and cannabinodivarin (CBND-C3) (**Figure 3**) whose structural elucidation required their total synthesis (Robert et al., 1977). CBND-C_5_ found in the plant’s flowers in low concentration, is considered a product of CBD photochemical conversion.

**FIGURE 4:**
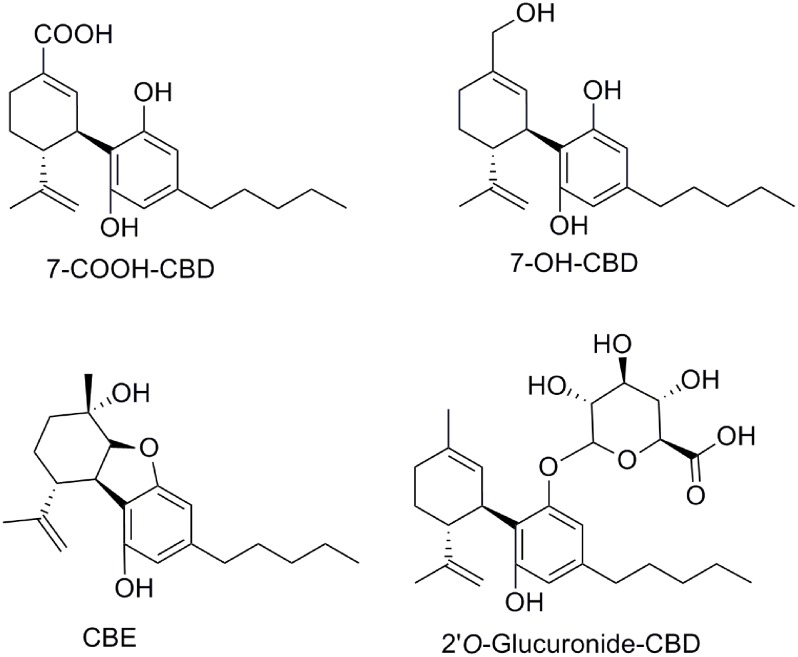
Selected metabolites of CBD.

The conversion of CBD into human metabolites has been the subject of a recent interesting review (Ujváry and Hanuš, 2016). CBD biotransformation shows considerable species variability. The main biotransformation, including hydroxylation and oxidation, involves the CYP450 enzyme family. While 7-hydroxy-CBD (7-OH-CBD) derivatives are found in low concentration, the most abundant metabolites are hydroxylated 7-carboxylic acid derivatives of CBD (7-COOH-CBD, **Figure 4**). Glucuronidation of CBD seems to frequently occur at the phenolic oxygen (**Figure 4**). Another cannabinoid metabolite, the so called cannabielsoin (CBE), has been identified in plants as a product of photo-oxidation from CBD and CBDA (Shani and Mechoulam, 1974; Ujváry and Hanuš, 2016), or by biotransformation using tissue cultures under normal growth conditions (Hartsel et al., 1983; Yamamoto et al., 1991). CBE was also identified as a metabolite in guinea pigs, mice, rabbits, and rats (Yamamoto et al., 1991). Despite the fact that CBD metabolites have been the subject of many studies, few *in vivo* studies have been published. Therefore, their therapeutic benefits remain to be established.

**FIGURE 5:**
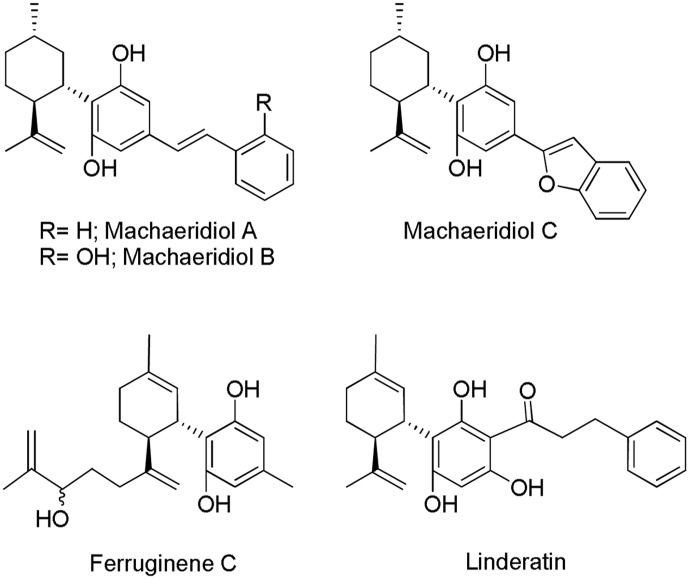
Cannabidiol (CBD)-related *Machaerium multiflorum*, *Rhododendron ferrugineum* L. and, *Lindera umbellata* Thunb. compounds.

Beyond the *Cannabis* plant, other naturally occuring products have been reported to interact with the ECS (Gertsch et al., 2010). However, only few of them are CBD-based compounds. Isolation and characterization of (+)-*trans*-hexahydrodibenzopyrans from the stem bark of the Amazonian liana *Machaerium multiflorum* Spruce led to the identification of the CBD related structures machaeridiols A, B, and C (**Figure 5**) (Muhammad et al., 2003). The total synthesis of these compounds via an efficient highly regio- and stereoselective approach has also been described (Huang et al., 2007). Although their activity at the CB_1_ and CB_2_ cannabinoid receptors has not been reported, these compounds displayed antimicrobial, antifungal, and antiparasitic activity in diverse *in vitro* assays (Muhammad et al., 2003). Machaeridiol B stands out as the most potent inhibitor against *Plasmodium falciparum* [chloroquine-sensitive (D6) and chloroquine-resistant (W2) clones] and *Leishmania donovani* with IC_50_ values in the low micromolar range (**Table 1**).

Ferruginene C, a methylpentanol derivative of CBD (**Figure 5**), was recently isolated from the leaves of *Rhododendron ferrugineum* L. as a mixture of diastereoisomers (Seephonkai et al., 2011). Ferruginene C has been shown to be cytotoxic in the HL-60 cancer cell-line (IC_50_ 13.7 μM) with selectivity toward non-cancerous cell-line. It binds weakly to CB_2_ and TPRV1 receptors, but it did not show significant affinity for CB_1_ and 5-HT_1A_ receptors (**Table 1**).

Even though linderatin (**Figure 5**), isolated from fresh leaves of *Lindera umbellata* Thunb. (Tanaka et al., 1984), is not considered a phytocannabinoid (Hanuš et al., 2016), it is interesting to include in the present review since closely related to CBD. No biological data have been reported so far.

## Synthetic Cbd Analogs

Due to the promising therapeutic effects of CBD in a wide variety of diseases, synthetic CBD derivatives have attracted the attention of drug discovery programs in both industry and academia with the aim to improve the potency, efficacy, or pharmacokinetic properties of this interesting phytocannabinoid.

**FIGURE 6:**
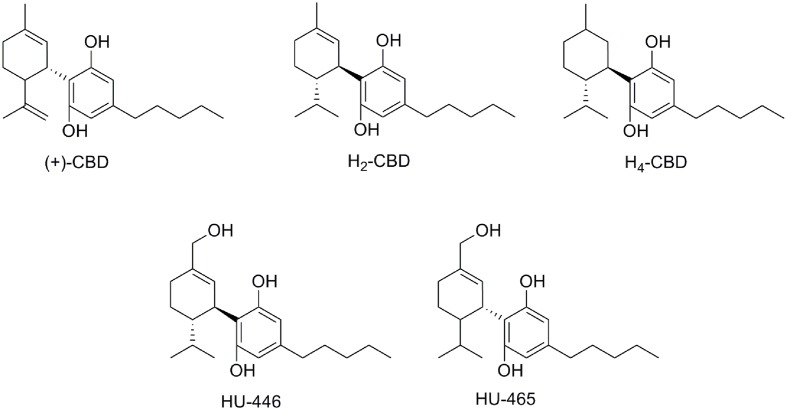
(+)-CBD and hydrogenated CBD derivatives.

Synthetic approaches for different CBD metabolites such as 7-COOH-CBD or 7-OH-CBD (**Figure 4**) have been reported (Tchilibon and Mechoulam, 2000; Mechoulam and Hanuš, 2002). Moreover, structural modifications on different pharmacophoric positions such as the lipophilic side chain, the phenolic hydroxyl groups or the C7-methyl have been widely accomplished. In addition to the (-)-CBD enantiomers, the (+)-CBD derivatives [(+)-CBD depicted in **Figure 6**] have also been synthesized and pharmacologically evaluated (Bisogno et al., 2001; Fride et al., 2004; Hanus et al., 2005). Measurements of the binding affinities of these compounds for the CB_1_ and CB_2_ cannabinoid receptors yielded unexpected outcomes. Contrary to the naturally occurring (-)-CBD analogs, which showed no orthosteric affinity, most of the compounds in the (+)-CBD series bind to both receptors displaying selectivity toward CB_1_ (**Table 1**).

Hydrogenation of CBD yielded the dihydro- and tetrahydro-cannabidiol derivatives H_2_-CBD and H_4_-CBD (**Figure 6**) (Ben-Shabat et al., 2006). Their effects on the production of reactive oxygen intermediates, nitric oxide, and tumor necrosis factor showed their anti-inflammatory capacity. In contrast to CBD, H_2_-CBD, and H_4_-CBD have affinity for the cannabinoid CB_1_ receptor (**Table 1**). Additionally, the (-)- and (+)-dihydro-7-hydroxy-CBD enantiomers (HU-446 and HU-465, **Figure 6**) have recently been synthesized and biologically characterized in an inflammatory model of encephalitogenic T cells (Kozela et al., 2015). Both compounds showed anti-inflammatory potential in inflammatory and autoimmune diseases models. However, only the (+)-enantiomer (HU-465) displays affinity for the cannabinoid CB_1_ and CB_2_ receptors (**Table 1**).

### 1′,1′ -Dimethylheptyl-CBD Derivatives

**FIGURE 7:**
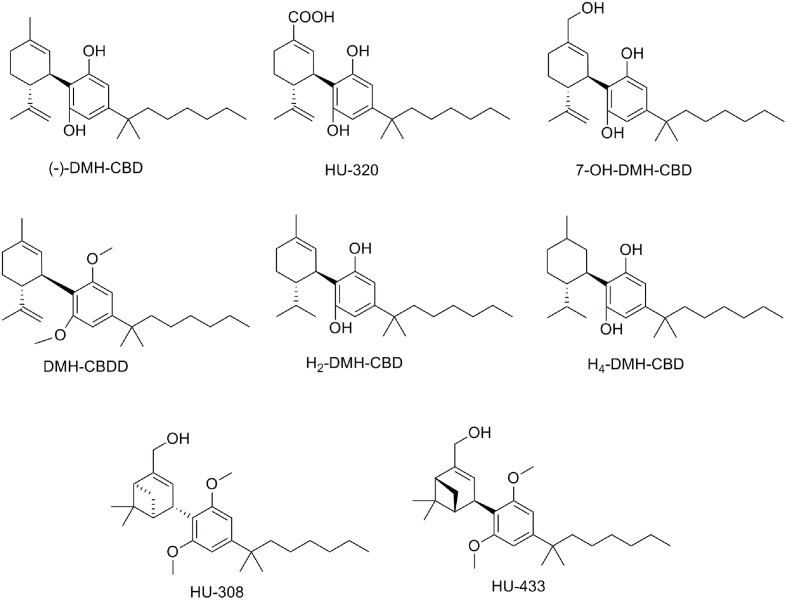
Dimethylheptyl (DMH)-CBD derivatives.

Taking into account that substitution of the pentyl chain of Δ^9^–THC by a 1′, 1′-dimethylheptyl (DMH) lipophilic alkyl chain resulted in more active compounds than natural Δ^9^–THC (Mechoulam et al., 1988), a similar approach was performed for the CBD scaffold (Mechoulam et al., 1990; Hanus et al., 2005) (**Table 1**). Thus, the synthesis of DMH-CBD derivatives, such as DMH-CBD, HU-320, DMH-CBDD, and 7-OH-DMH-CBD (**Figure 7**) have been reported by Mechoulam and coworkers (Leite et al., 1982; Hanus et al., 2005). Introduction of the DMH alkyl chain in the (-)-DMH-CBD series did not change the lack of CB_1_ and CB_2_ receptor affinity except for (-)-7-OH-DMH-CBD that moderately binds to CB_2_ (**Table 1**) (Bisogno et al., 2001). However, in the case of the (+)-DMH-CBD series, the presence of the DMH alkyl chain improved both CB_1_ receptor affinity compared to (+)-CBD (**Table 1**). (-)-DMH-CBD analogs have displayed anxiolytic, analgesic, anti-inflammatory, or antiproliferative effects in diverse assays (Burstein, 2015). For instance, (-)-DMH-CBD has shown anti-inflammatory and antiproliferative properties in human acute myeloid leukemia, microglial or encephalitogenic T cells (Juknat et al., 2016). The carboxylic acid HU-320 produced strong anti-inflammatory and immunosuppressive effects in an *in vivo* model of collagen-induced arthritis (Sumariwalla et al., 2004). Interestingly, (-)-7-OH-DMH-CBD exhibited potent inhibition of electrically evoked contractions of the mouse vas deferens that was not mediated through CB_1_, CB_2_, TRPV1, opioid, or α_2_-adrenergic receptors (Fride et al., 2005; Pertwee et al., 2005).

As previously mentioned for the pentyl CBD derivatives, hydrogenation of DMH-CBD has been studied (Ben-Shabat et al., 2006). Partial hydrogenation gave H_2_-DMH-CBD (**Figure 7**) as the major epimer (hydrogenation at C8) with small amounts of the hydrogenated C1 epimer being obtained. Full hydrogenation allowed the formation of H_4_-DMH-CBD (**Figure 7**). These hydrogenated compounds, which bind to the CB_1_ receptor with affinity constants in the nanomolar range, displayed weak anti-inflammatory effects when compared to CBD or DMH-CBD.

The pinene dimethoxy-DMH-CBD derivative HU-308 (**Figure 7**) was identified decades ago as a potent peripheral CB_2_-selective agonist (Mechoulam et al., 1990; Hanus et al., 1999). HU-308 has shown very interesting properties such as anti-inflammatory, analgesic, neuroprotective or antitumor effects, and has been used as a pharmacological tool in numerous cannabinoid studies contributing to the progress in this field (e.g., Hanus et al., 1999; Ofek et al., 2006; Rajesh et al., 2007a,b; Burstein, 2015). More recently, the efficacy of HU-308 and HU-433, two enantiomers, has been tested in ovariectomy-induced bone loss and ear inflammation (Smoum et al., 2015) showing an inverse relationship between binding affinity and biological potency.

### Other Modifications on the C4′-Alkyl Chain

**FIGURE 8:**
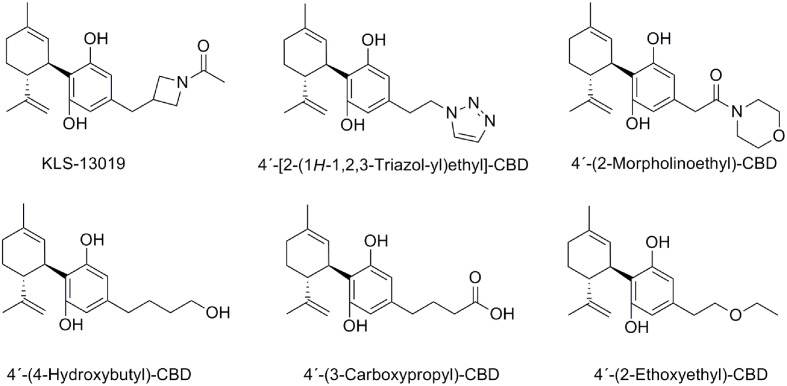
Cannabidiol analogs modified on the C4′-alkyl chain.

In order to improve oral bioavailability and solubility issues, a novel series of CBD analogs have recently been synthesized (Kinney et al., 2016) (**Figure 8**). Structural modifications at the pharmacophoric lipophilic chain allowed fine-tuning the “drug-likeness” of this scaffold by variation of different physicochemical parameters such as the number of hydrogen bond donors, acceptors, and polar surface area. Among these new derivatives depicted in **Figure 8**, KLS-13019 stands out as being 50-fold more potent and more than 400-fold safer than CBD preventing damage to hippocampal neurons induced by ammonium acetate and ethanol with improved oral bioavailability compared to CBD (Kinney et al., 2016).

### Halogenated CBD Derivatives

**FIGURE 9:**
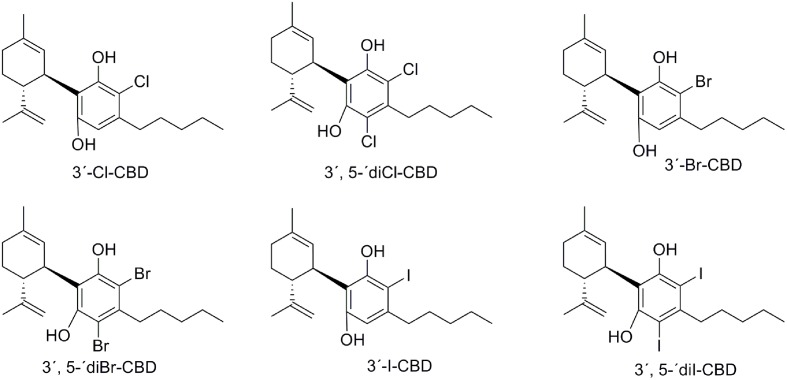
Chlorinated, brominated, and iodinated CBD derivatives.

Structural modifications of CBD include halogenated substituents on the phenol ring. The first reported halogenations occurred at the 3′ and/or 5′ positions by chlorine, bromine or iodine substitution, allowing the preparation of 3′-Cl-CBD, 3′,5′-diCl-CBD, 3′-Br-CBD, 3′,5′-diBr-CBD, 3′-I-CBD, and 3′,5′-diI-CBD (**Figure 9**) (Usami et al., 1999). These halogenated compounds were evaluated in murine models of barbiturate-induced sleep prolongation, electroshock-induced seizures and locomotor activity resulting in activity similar to CBD for the monohalogenated analogs, whereas the dihalogenated derivatives displayed lower activity (**Table 1**).

**FIGURE 10:**
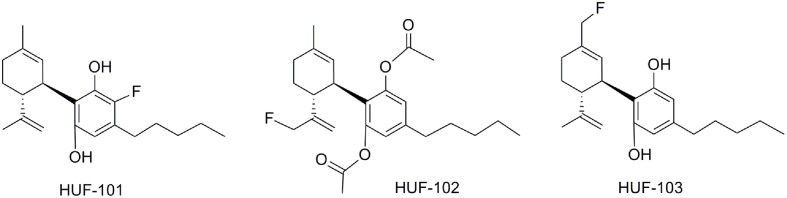
Fluorinated CBD derivatives.

The synthesis and pharmacological evaluation of three new fluorine halogenated CBD derivatives have been reported (Breuer et al., 2016). Two of these were fluorinated at the cyclohexenyl ring substituent (**Figure 10**: HUF-102 and HUF-103), and the third one was fluorinated at the phenol ring (HUF-101). HUF-101 displayed the most promising results in four mice behavioral assays (elevated plus-maze, forced swimming test, prepulse inhibition, and marble burying test) that target anxiolytic, antidepressant, antipsychotic and anticompulsive activity respectively. HUF-101 may be an interesting prototype for further development since it showed higher potency than CBD in the animal assays cited above. In these tests, HUF-102 did not show activity at the doses tested (1–10 mg/kg), whereas HUF-103 showed moderate to low activity compared to HUF-101.

### Modifications on the Hydroxyl Groups

**FIGURE 11:**
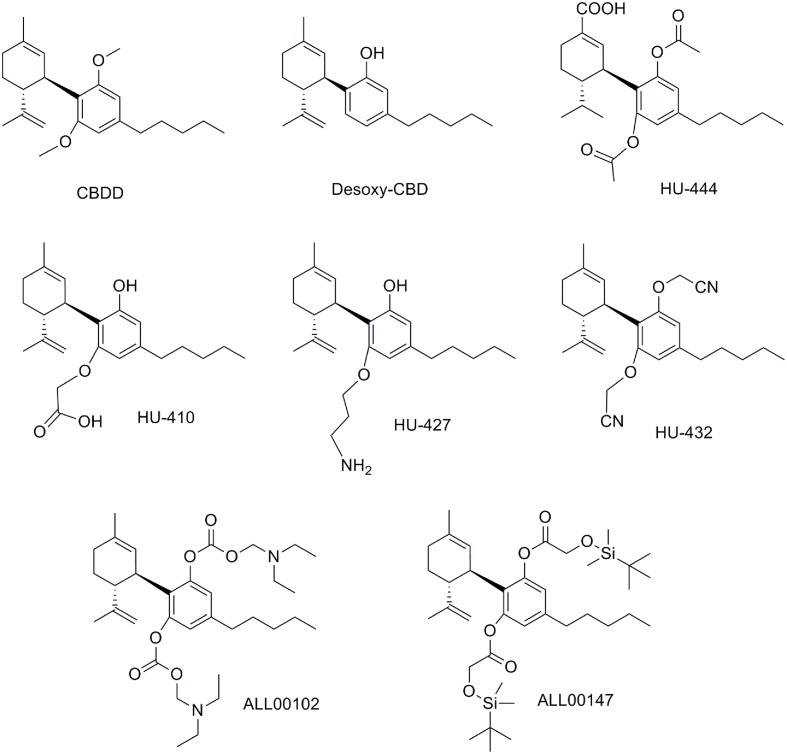
Cannabidiol derivatives modified at the hydroxyl groups.

Modifications on the resorcinol hydroxyl groups have been explored. Computational studies suggested that the removal of one of the CBD hydroxyl groups may enable the ligand to reach the CB_1_ binding site (Reggio et al., 1995). Thus, desoxy-CBD represented in **Figure 11** was synthesized and evaluated. Pharmacological data for desoxy-CBD corroborated the computational studies showing CB_1_ partial agonism in the mouse vas deferens assay.

Different research groups have developed acetylations and alkylations at one or both phenolic hydroxyls. For instance, the dimethylated CBD derivative named CBDD (**Figure 11**), as well as the monomethylated derivative (CBD-2′-monomethylether o *O*-methylcannabidiol) revealed higher potency and selectivity as 15-lipoxygenase inhibitors compared to CBD (Takeda et al., 2009, 2011). Consequently, the resorcinol moiety seems to be a determinant for the activity in this target. Further studies performed with CBDD suggest that this compound is not only a potential prototype for atherosclerosis treatment, but also a pharmacological tool to study the mechanisms of body weight regulation (Takeda et al., 2015). Other alkylations on the phenolic hydroxyl group have been reported such as *O*-propyl- and *O*-pentylcannabidiol that have been structurally characterized but no pharmacological data have been described so far (Hendricks et al., 1978).

Cannabidiol derivatives bearing one or both hydroxyl substitutions have been reported in the patent literature to be active as anti-inflammatory agents (Mechoulam et al., 2008). Selected examples disclosed in this patent (HU-410, HU-427, and HU-432) are depicted in **Figure 11**. It is interesting to highlight that some of these compounds present improved solubility, stability and bioavailability parameters when compared with CBD. Likewise, the non-CB_1_, non-CB_2_ ligand HU-444 has shown anti-inflammatory properties *in vitro* and *in vivo* in a murine model of collagen-induced arthritis (Haj et al., 2015).

**FIGURE 12:**
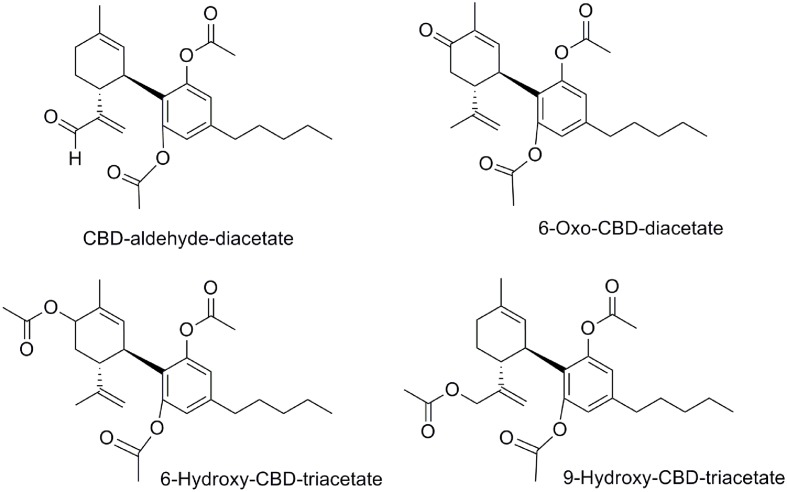
Diacetylated-CBD analogs.

In addition, the *in vivo* anticonvulsant activity of four diacetylated-CBD analogs (CBD-aldehyde-diacetate, 6-oxo-CBD-diacetate, 6-hydroxy-CBD-triacetate, and 9-hydroxy-CBD-triacetate, **Figure 12**) was demonstrated in a mouse model (Carlini et al., 1975). Their effects against maximal electroshock convulsions, potentiation of pentobarbital sleeping-time and reduction of spontaneous motor activity were evaluated obtaining significant anticonvulsant effects at high doses. It is noteworthy that the safety, efficiency, and potency of these four compounds were lower than that of CBD in the same assays.

At that point it is interesting to mention that these diacetate CBD derivatives could have been considered as prodrugs. Considering that CBD is rapidly distributed in adipose tissues and it undergoes a CYP3A- and CYP2C- dependent first-pass metabolism to give 7-hydroxy-CBD (Fasinu et al., 2016), a prodrug concept could be very useful. Therefore, the phenyl acetate groups could be deacetylated to give CBD. The pharmaceutical company, AllTranz, now called Zyberba Pharmaceutics, developed transdermal solutions of CBD-esters and -carbonates among others. The dicarbonate All00102 and the diglicolate AL00147 shown in **Figure 11** are two examples disclosed in a AllTranz’s patent (Stinchcomb et al., 2009). Another company, Kalytera Therapeutics is currently undertaking the preclinical stage of K-1012, a bi-phosphate derivative of CBD designed as a prodrug indicated for acute respiratory distress syndrome.^2^

### Quinone Derivatives of CBD

The quinone derivative of CBD, HU331, was first synthesized in Mechoulam et al. (1968) by oxidation of CBD. HU331 has been suggested to be a CBD metabolite having inhibitory effect on cytochrome P450 (Bornheim and Grillo, 1998). It was not until Kogan et al. (2004) that the antineoplastic activity of HU-331 was reported. HU-331 was very effective in reducing growth of human colon carcinoma HT-29 cells in nude mice. The mechanism by which HU-331 acts as an antitumor agent is independent of the CB_1_ and CB_2_ cannabinoid receptors. HU-331 does not promote cell death via cell cycle arrest, cell apoptosis, or caspase activation. Extensive studies have shown that HU-331 anticancer properties were due to selective inhibition of the ATPase function of human topoisomerase IIα (Kogan et al., 2007; Peters and Kogan, 2007; Regal et al., 2014). Thus, HU-331 with a selective topoisomerase inhibition is expected to have less off-target toxicity than doxorubicin which antitumor activity is mediated through numerous mechanisms, such as apoptosis, abrogation of the cell cycle, activation of caspases, generation of ROS, and inhibition of both topoisomerases among others.

Structural modifications realized on the substituents of HU-331 led to the benzoquinones having anti-proliferative activity against diverse cancer cell lines (Petronzi et al., 2013). Unlike HU-331, benzoquinone mechanism of action involves caspase activation, poly-(ADP-ribose)-polymerase (PARP) protein cleavage, and reactive oxygen species (ROS) production. These data show the influence of CBD structure compared to the quinone core on the processes producing anticancer effects.

**FIGURE 13:**
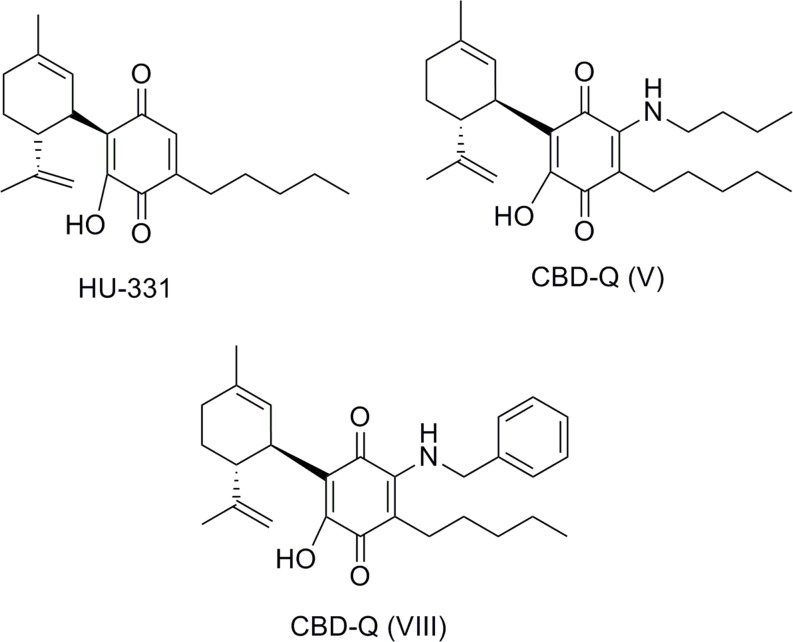
Quinones related to CBD.

A recent patent from VivaCell Technology discloses HU-331 analogs which act as PPARγ agonists showing a neuroprotective profile in different models (Appendino et al., 2015). The disclosed quinones are substituted in position 3′ by different amines or carboxylates that were synthesized by amination of CBD or esterification of CBDA respectively. Compounds CBD-Q (V) and CBD-Q (VIII) illustrated in **Figure 13** are representative of the HU-331 analogs.

### Miscellaneous CBD Derivatives

**FIGURE 14:**
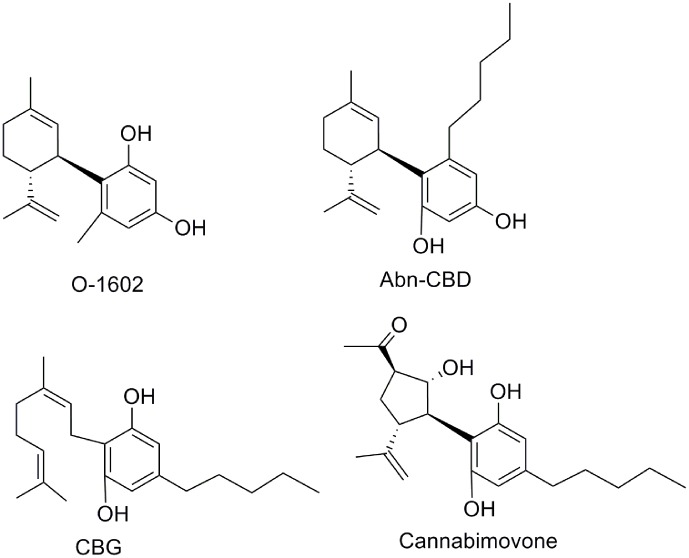
Miscellaneous CBD derivatives.

Abnormal cannabidiol (Abn-CBD) (Razdan et al., 1974), a non-psychoactive synthetic regioisomer of CBD (**Figure 14**), has been the subject of numerous studies that have shown Abn-CBD therapeutic potential as a vasodilator (Johns et al., 2007), antibacterial (Appendino et al., 2008), antidiabetic (McKillop et al., 2016), or anti-colitis agent (Krohn et al., 2016). Recently, two molecular targets, GPR55 and GPR18, have been identified for Abn-CBD (Johns et al., 2007; Ryberg et al., 2007; Console-Bram et al., 2014). Abn-CBD stimulated [^35^S]GTPγS binding at GPR55 (Oka et al., 2007) and increased calcium mobilization and ERK1/2 phosphorylation at GPR18 (Console-Bram et al., 2014).

The synthetic cannabinoid O-1602 that does not bind significantly to CB_1_ or CB_2_ receptors, stimulates GTPγS activation in membranes from human recombinant GPR55-expressing cells (EC_50_ = 1.4 nM) (Johns et al., 2007; Console-Bram et al., 2014). *In vivo*, O-1602 showed anti-inflammatory activity in mice with cerulein-induced acute pancreatitis characterized by an increased expression of GPR55 receptor (Li et al., 2013). O-1602 has also been shown to increase levels of GPR18-mediated MAPK activity and calcium mobilization, but not β-arrestin signaling, thus supporting that O-1602 acts as a biased-agonist at GPR18 (Console-Bram et al., 2014). Data have been reported suggesting the therapeutic potential of O-1602 for diseases related to the central nervous system (Ashton, 2012), or to metabolic diseases (Romero-Zerbo et al., 2011).

Another minor component of *Cannabis sativa* is cannabigerol (CBG) (Gaoni and Mechoulam, 1971). Structurally, CBG can be considered the cyclohexenyl-opened analog of CBD. Different therapeutic applications have been proposed for CBG, more recently CBG has been shown to have antibacterial action (Appendino et al., 2008), antidepressant-like action (El-Alfy et al., 2010), and anti-inflammatory properties for bowel disease (Borrelli et al., 2013). Molecular targets of CBG include the α_2_ adrenergic receptor, TRP channels, cyclooxygenase (COX-1 and COX-2) enzymes, as well as the 5-HT_1A_ and cannabinoid receptors (Cascio et al., 2010; De Petrocellis et al., 2011; Ruhaak et al., 2011). Cannabimovone is one of the latest natural phytocannabinoids that has been extracted from a cultivar of hemp rich in CBD (Taglialatela-Scafati et al., 2010). The terpenoid structure of cannabimovone replaces the cyclohexenyl ring of CBD by a functionalized cyclopentane including four contiguous stereocenters. Its total synthesis has been reported very recently (Carreras et al., 2016). Cannabimovone is devoid of CB_1_ and CB_2_ activity, whereas it is a weak TPRV1 agonist.

## Conclusion

A significant amount of preclinical data has shown the high therapeutic potential of CBD especially in inflammatory mouse models. According to ClinicalTrials.gov records, CBD is currently tested in clinical phases for different inflammatory diseases. The results of the first clinical study of CDB for the treatment of inflammatory bowel have been published very recently. Unfortunately, the effects of CBD on Crohn’s disease were ineffective in a randomized placebo-controlled trial on 20 patients probably due to low used doses (Naftali et al., 2017). The potential antiepileptic effects of CBD in patients suffering seizures associated with Lennox–Gastaut syndrome and in children and young patients with Dravet syndrome are currently on-going. The research has tended to focus on CBD therapeutic applications. Less attention has been paid to the therapeutic utility of CBD derivatives.

Despite the identifications of CBD metabolites and naturally occurring CBD analogs, in general, their pharmacological properties have not been extensively studies. In what concerns synthetic CBD-based compounds, several of them have shown interesting pharmacological properties but none has been introduced into clinical trials yet. In a pharmacological point of view, whereas CBD does not have affinity for both classical CB_1_ and CB_2_ cannabinoid receptors, most of (+)-CBD derivatives do bind to CB_1_ and/or CB_2_ receptors. Others, such as Abn-CBD, O-1602, CBG, cannabimovone, ferruginene C, (-)-CBDV, and (-)-CBDA, have shown activity at other receptors including TPRV1, GPR35 and/or GPR18 receptors, or enzymes such as COX-2. A limitation of the development of CBD synthetic derivatives probably resides in the lack of a unique common molecular target.

In future therapeutic development of CBD derivatives, it will be prudent to take into account some structural considerations around the CBD scaffold. One of them is the possible atropisomerism around the phenyl–hexenyl bond. Ortho-substitution on the phenyl ring could have stereochemical consequences generating hindered rotation of the phenyl–hexenyl bond due to steric or electronic constraints, generating two isolable conformers in the case of slow interconversion (Berber et al., 2014; Flos et al., 2016). Thus, it is necessary to consider the implication of a possible atropoisomerism for new CBD analogs discovery (Clayden et al., 2009).

The complexity of the pharmacological processes of CBD and CBD analogs suggest that a better understanding of their mechanism of action is required to devise successful synthetic CBD-based drug therapies.

## Author Contributions

PM, PHR, and NJ substantially contributed to the redaction of the manuscript. Then, they all approved the manuscript to be published.

## Conflict of Interest Statement

The authors declare that the research was conducted in the absence of any commercial or financial relationships that could be construed as a potential conflict of interest.

## Footnotes

1 ClinicalTrials.gov. A Study of GWP42006 in People With Focal Seizures. https://clinicaltrials.gov/ct2/show/NCT02369471 (accessed September 22, 2016).

2 https://kalytera.co/programs/preclinical/ (accessed June 8, 2017).
